# Laser-Inscribed Glass Microfluidic Device for Non-Mixing Flow of Miscible Solvents

**DOI:** 10.3390/mi10010023

**Published:** 2018-12-29

**Authors:** Valeria Italia, Argyro N. Giakoumaki, Silvio Bonfadini, Vibhav Bharadwaj, Thien Le Phu, Shane M. Eaton, Roberta Ramponi, Giacomo Bergamini, Guglielmo Lanzani, Luigino Criante

**Affiliations:** 1 Center for Nano Science and Technology, Istituto Italiano di Tecnologia, 20133 Milano, Italy; valeriaitalia.vi@gmail.com (V.I.); silvio.bonfadini@iit.it (S.B.); guglielmo.lanzani@iit.it (G.L.); 2 Dipartimento di Fisica, Politecnico di Milano, Piazza Leonardo da Vinci 32, 20133 Milano, Italy; argyrogiak@gmail.com (A.N.G.); thien.lephu@polimi.it (T.L.P.); roberta.ramponi@polimi.it (R.R.); 3 Istituto di Fotonica e Nanotecnologie-Consiglio Nazionale delle Ricerche (IFN-CNR), Piazza Leonardo da Vinci 32, 20133 Milano, Italy; vibhavbharadwaj@gmail.com; 4 Department of Chemistry Giacomo Ciamician University of Bologna Via Selmi 2, I-40126 Bologna, Italy; giacomo.bergamini@unibo.it

**Keywords:** optofluidics, lab-on-a-chip, femtosecond laser, laser micromachining, diffusion

## Abstract

In recent years, there has been significant research on integrated microfluidic devices. Microfluidics offer an advantageous platform for the parallel laminar flow of adjacent solvents of potential use in modern chemistry and biology. To reach that aim, we worked towards the realization of a buried microfluidic Lab-on-a-Chip which enables the separation of the two components by exploiting the non-mixing properties of laminar flow. To fabricate the aforementioned chip, we employed a femtosecond laser irradiation technique followed by chemical etching. To optimize the configuration of the chip, several geometrical and structural parameters were taken into account. The diffusive mass transfer between the two fluids was estimated and the optimal chip configuration for low diffusion rate of the components was defined.

## 1. Introduction

The recent introduction of microfluidics in chemistry and biology has led to a paradigm shift in both fields. Lab-on-a-Chip is now a commonly known concept and significant efforts have been made for the realization of multifunctional integrated systems for chemical analysis [1], cell culture, and biochemical systems investigation [2], but most importantly for multiphase chemical reactions even for miscible solutions. Microfluidic reactors have already been proven valuable due to their high surface-to-volume ratio, the scale-out capabilities for industrial applications, the higher yield over batch reactors, and the versatility of the microfluidic chip set-ups [3]. By manufacturing a microfluidic chip with a suitable geometry, it is possible to manage simultaneously two or more fluids and create dynamic interfaces between them while avoiding active mixing due to laminar flow [4,5]. The present approach exploits (mimics) liquid–liquid interfaces which could not be accessed in batch situations. Diffusive mixing between two interfacing laminar flows is a theoretically, and in some cases experimentally, well-defined process [6,7]. It depends mainly on the area of interaction, the laminarity of the flow, the time of interaction, and the concentration gradient between two or more streams. 

A wide variety of materials and techniques have been employed for the fabrication of microfluidic systems [8]. The most popular process is the soft lithographic fabrication of 2D chips on polydimethylsiloxane (PDMS) [9] and other elastomers. The technique is easy, fast, and has a low cost, but although PDMS’ porosity is a virtue for long-term cell cultures, it becomes a drawback when it comes to chemical analysis or organic synthesis because it cannot be defined as chemically inert. PDMS can undergo swelling due to solvent adsorption in the pores, creating deformations on the microfluidic channels [10]. Extensive deformation can create leakages, thus compromising the chip. Combined with the incompatibility with many organic solvents, PDMS has a limited range of applications in synthetic chemistry. 

Even though a plethora of water-based biological applications have been demonstrated for PDMS microfluidic chips, the use of other solvents is prohibited due to the incompatibility of PDMS. It has been demonstrated that water-based chemical reactions [4] are able to be performed on the interface of two interacting laminar flows in a PDMS microfluidic chip. This high yield and recyclable approach could also be applied in organic chemistry, especially for reactions that involve toxic or expensive reagents. Lee et al. [10] performed an extensive study on the compatibility of PDMS with a variety of organic solvents, and it is evident that, for a broader application of microfluidics in organic chemistry, PDMS is not the optimal material. One of the most promising materials that can overcome the above mentioned challenges is fused silica.

Fused silica microfluidic devices have been well established, but the exploitation of a liquid–liquid interface that is present in a Y-shaped fluidic system requires an extensive study on the behavior of pressure-driven flows. Glass chips differ from PDMS as they are rigid, and they do not exhibit deformation upon high-pressure-driven flow [11]. Fused silica is compatible with a wide variety of organic solvents as well as water, and since it is not gas-permeable, it can be used as a material for the fabrication of microreactors for a wide variety of reactions, including water splitting for hydrogen production.

In this paper, we report the fabrication and characterization of a double Y-branch fused silica microfluidic device for the introduction, interaction, and separation of two miscible solutions characterized by laminar flow, taking advantage of the femtosecond laser irradiation followed by the chemical etching (FLICE) method [12,13,14,15,16]. There are many advantages attributed to this microfabrication technique in comparison with traditional photolithography, including the ability to quickly realize 3D monolithic structures completely buried in the substrate without the requirement of masks or a clean room. With FLICE, there is no need to create complex microfluidic chips in two halves to be subsequently welded as it is a process which often leads to sealing problems. As a preliminary investigation, the laser-fabricated, fused silica microfluidic device was used for the study of the diffusion of Rhodamine 6G (R6G) in the ethanol–ethanol interface. The angle between the two inlets and the height of the chamber were varied, and the diffusion was qualitatively determined for different flow rates. R6G was used as a colorant for one of the two streams due to its optical properties and its well-established diffusivity in ethanol.

## 2. Materials and Methods

### 2.1. Methods

Fused silica glass is ideally suited for this application as it possesses several important characteristics: it is chemically inert to a variety of solvents, hydrophilic, thermally and mechanically stable, and optically transparent in a wide range of wavelengths [12,17]. To fabricate the optofluidic device in the bulk of glass, we exploit the FLICE method [12,13], which requires two steps: (1) tightly focused, femtosecond, laser pulses drive a permanent and localized periodic redistribution of material density, which defines the desired structure on the surface or in the bulk of fused silica [18]; (2) chemical etching of the laser-modified volume by a strong acid or a strong base (typically HF or KOH, respectively) to remove the irradiated zone, producing the hollowed-out, microfluidic device [15].

**Figure 1:**
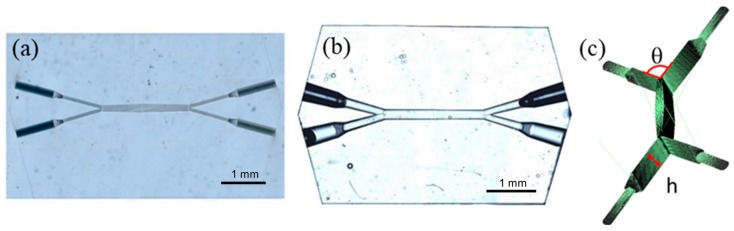
Microfluidic chip geometry; image from optical microscope of the chip after (**a**) femtosecond (fs) laser irradiation and subsequent; (**b**) chemical etching; (**c**) schematic of chip design, where h is the chamber height and *θ* is the separation angle.

The femtosecond laser used for device fabrication in fused silica was a generatively amplified Yb:KGW system (Pharos, Light Conversion, Vilnius, Lithuania) with 230-fs pulse duration, 515-nm wavelength (frequency doubled), and 500-kHz repetition rate focused with a 0.42-NA microscope objective (M Plan Apo SL50X Ultra-Long Working Distance Plan-Apochromat, Mitutoyo, Kawasaki, Japan). Computer-controlled, 3-axis motion stages (ABL-1000, Aerotech, Pittsburgh, PA, USA) interfaced by CAD-based software (ScaBase, Altechna, Vilnius, Lithuania) with an integrated acousto-optic modulator were used to translate the sample relative to the laser irradiation desiderate patch. An average power (pulse energy) of 200 mW (400 nJ) and a scan speed of 5 mm/s were used to laser-pattern the microfluidic device shown in Figure 1a. A multiscan writing procedure with 7 μm spacing between transverse scans was adopted to form the microfluidic device. The thickness of the fused silica windows was 1 mm, and the buried microfluidic chips were laser-inscribed at a depth of 0.5 mm. The overall fabrication time of a single chip varied between 57 min and 69 min for chamber heights between 100 μm and 500 μm. The sample was etched in a sonication bath of HF (20% vol in water), with a feedback-controlled temperature of 37 °C. The etching rate of the laser-exposed area of the fused silica was 500 μm/h, whereas for the non-exposed area, the etching rate was 20 μm/h. The resulting rectangular chamber’s internal dimensions were 2 mm × 200 μm (length × width) with a height *h* that varied from 100 μm to 500 μm (Figure 1c).

The suitable structure for the diffusion study in a microfluidic chip is the double Y configuration as shown in Figure 1, showing optical microscopic images of the device after femtosecond laser irradiation (Figure 1a) and then after chemical etching (Figure 1b). The final microfluidic device consists of two inlets and two outlets at both ends of a long chamber in which the interface interaction occurs.

To complete the characterization of the chip, the behavior of diffusive mass transfer was studied by varying the separation angle between the inlet/outlet channels, *θ*, and the pumping pressure of the fluids, *p*. To avoid turbulence, the inlet tubes and the interaction chamber were designed considering the continuity of the fabrication process and the equality in resistance for both the inlets and outlets of the chip. A microfluidic pump (OB1, Elveflow, Paris, France) was connected to the reservoirs of the solutions. Polytetrafluoroethylene (PTFE) tubing was inserted into the reservoirs and drove the fluids into the chips by Polyether ether ketone (PEEK) tubing with an outer (inner) diameter of 360 μm (150 μm) by using an appropriate adapter. The latter tubing was connected to the glass chip using ultraviolet (UV) glue. The materials for both tubings as well as the reservoirs and the glue were selected due to their extraordinary chemical inertness to organic solvents.

### 2.2. Materials

To visualize the interaction zone between the two parallel flows, an optical technique was used. Using a coloured (1 mM Rhodamine 6G in ethanol) and a transparent solution (pure ethanol) it was possible to understand how the geometric and microfluidic parameters influence the diffusive mass transfer between two interfacing laminar flows.

The dye solution was prepared starting from R6G powder (Sigma-Aldrich) dissolved in filtered ethanol. The solution was filtered once more to prevent undissolved dye particles and impurities from entering the chip and creating turbulence. The Rhodamine solution was stored in glass vials in a dry, cool, and dark environment to prevent degradation.

## 3. Results

### 3.1. Data Analysis

**Figure 2:**
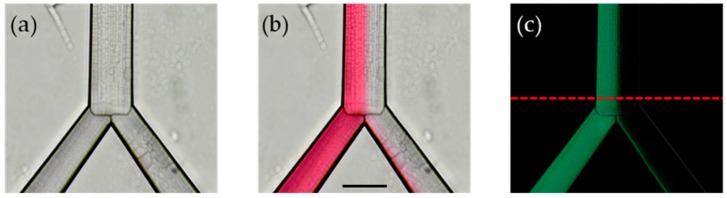
Visual analysis technique of laser-fabricated microfluidic chip: (**a**) reference image, (**b**) colored image with the flowing dye solution; (**c**) negative image obtained by subtracting (**a**) from (**b**). The red dashed line indicates the output of the chip position chosen to extract the intensity profile. Scale bar corresponds to 200 μm.

Starting from the work of Werts et al. [7], we developed a simple and useful method to extract and analyze data using a conventional optical microscope. We obtained an empty channel image to use as a reference (Figure 2a), and subsequently we imaged the flowing colorants, varying the pumping pressure (Figure 2b). Using a custom image management algorithm (developed in MATLAB), the two aforementioned data images can be subtracted to indicate the difference i.e., the dye solution flow, and to eliminate electronic noise caused by the intrinsic roughness of the device. The visible roughness of the chip, as well as the discontinuation between the chamber and the outlets, in Figure 2a,b is caused by the laser writing pattern of the device, and even though it can be improved by post-fabrication annealing, there was no visible turbulence in the chip due to this effect. As it has been reported in the past, the roughness of microchannels formed by this technique have sub-micrometer roughness [19].

**Figure 3:**
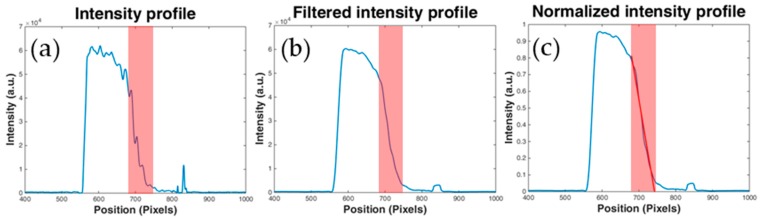
Intensity profile of the flow behavior processed with MATLAB algorithm. The x-axis represents the position in the image [pixels], while the y-axis is the fluorescence intensity in arbitrary units (a.u.). The red region indicates the diffusion zone between the two liquids. (**a**) original, (**b**) processed by a low-pass filter, and (**c**) normalized intensity profile. The slope of the red line represents the diffusion behavior.

In this way, we obtained a clear negative image (Figure 2c) from which the cross-section intensity profile is extracted at the fixed position near the outlet of the channel (red dashed line in Figure 2c). The typical intensity profile of the bi-colour image (Figure 2c) is shown in Figure 3a. The signal is then processed with a low-pass filter (Figure 3b) and normalized (Figure 3c) to be comparable with other intensity profiles.

At this point, in order to qualitatively define the conditions for the lowest diffusion of R6G inside the channel, we calculated the slope of the cross-section intensity profile with linear interpolation. The left region of high intensity in the normalized profile in Figure 3c indicates the presence of the dye, while the region of low intensity on the right indicates the absence of it. The transitional region highlighted in red between the high and low intensity regions represents the diffusion zone. It is important to note that the value of the slope in the diffusion region is inversely proportional to the amount of diffused dye between the liquids (ideally infinite slope indicates zero diffusion).

### 3.2. Preliminary Considerations

Reynolds number (Re) and Peclet number (Pe) are two dimensionless values that define fluidic and diffusive mixing characteristics of a microfluidic system, respectively. They are described as [20,21]

(1) Re=ρυ¯lμ,  Pe=υ¯lD

where, ρ is the density, $\bar{\upsilon }$ is the mean flow velocity, *μ* is the dynamic viscosity of the fluid, l is the characteristic length of the microfluidic channel, and *D* is the diffusivity. A flow with Re lower than 2300 is considered laminar, while a high Pe number defines the number of channel widths required to completely mix two fluids by diffusion.

For ethanol, ρ = 789 kg/m^3^ and *μ* = 1.2 mPa·s. For Rhodamine 6G, *D* = 3 × 10^−^^10^ m^2^/s [22]. The characteristic length of the rectangular channel was calculated to be 1.33 × 10^−4^ m for *h* = 100 μm and 2.85 × 10^−4^ m for *h* = 500 μm.

In a microfluidic device, we can assume the Hagen–Poiseuille equation to describe the relationship between the pressure drop (Δ*P = P*_in_ *– P*_out_) and the flow rate (*Q*) of pressure-driven flow [23]:

(2) $$\Delta P_{\mathrm{total}}=\;R_{\mathrm{total}}Q=R_{\mathrm{total}}\bar{\upsilon }S$$

where *R* is the hydrodynamic resistance of the microfluidic system and *S* is the cross-sectional area of the microfluidic chamber. For a single microfluidic chip, we can consider *R* and *S* as constants, meaning that the fluid velocity increases with the pumping pressure.

Starting from Stokes equations, it is possible to calculate the fluidic resistance for a fluid with viscosity *μ* flowing inside a [23]:

- cylindrical channel (tubings) with length *L* and internal radius *r*,
  (3) $$R_{\mathrm{tubing}}=\frac{8\mu L}{\pi r^{4}}$$
- rectangular channel (glass chip) with length *L*, height *h*, and width *w*,
  (4) $$R_{\mathrm{chip}}=\frac{12\mu L}{1-0.63\left(\frac{h}{w}\right)}\cdot \frac{1}{h^{3}w}$$

In the present work, since all the elements are connected in series, we can consider $R_{\mathrm{total}}=R_{\mathrm{tubing}}+R_{\mathrm{chip}}$. Considering that $R_{\mathrm{tubing}}\gg R_{\mathrm{chip}}$ due to the tubing’s comparable radius to the chip but a comparatively much greater *L*, we can neglect the resistance of the glass chip and calculate from Equation (3) that $R_{\mathrm{total}}\;$= 1.11 × 10^11^ mbar∙s/m^3^ for *r* = 75 μm and *L* = 11.5 cm. Assuming that the pressure given by the pump is equal to $\Delta P_{\mathrm{total}}$, we can calculate the flow rate and subsequently, the flow velocity of the chips with different *h* and varying pumping pressures, as reported in Table 1.

### 3.3. The Effect of Pumping Pressure

**Figure 4:**
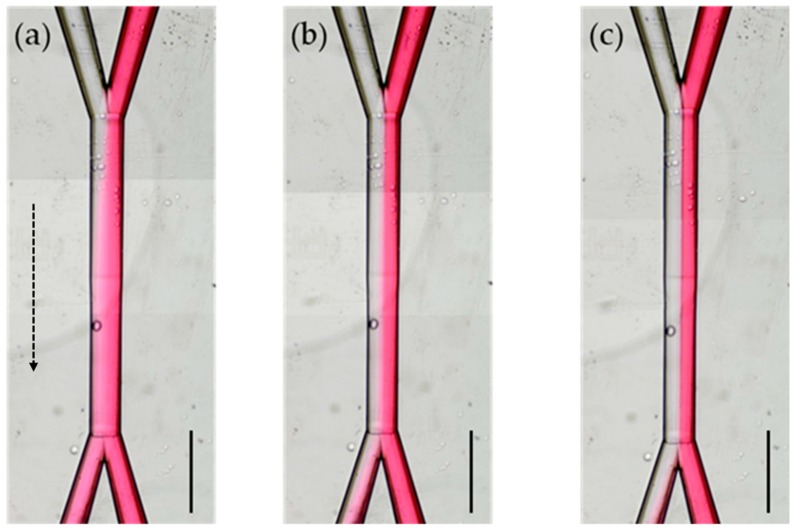
Diffusion behavior inside a microfluidic chip with 30° incident angle and *h* = 500 μm, at increasing pumping pressure (**a**) Δ*P* = 25 mbar; (**b**) Δ*P* = 75 mbar; (**c**) Δ*P* = 200 mbar. Scale bars correspond to 500 μm. Arrow indicates the flow direction.

The flow velocity is directly dependent on the pumping pressure according to the Hagen–Poiseuille equation in Equation (2), and it is the only parameter other than the geometrical characteristics of the chip that can affect the diffusion of R6G in ethanol. Diffusion is a time-dependent process, and it is obvious that a slowly flowing solution (i.e., in the case of Δ*P* = 25 mbar) exhibits greater diffusion, as seen in Figure 4.

### 3.4. The Effect of Angle Between Inlets

**Figure 5:**
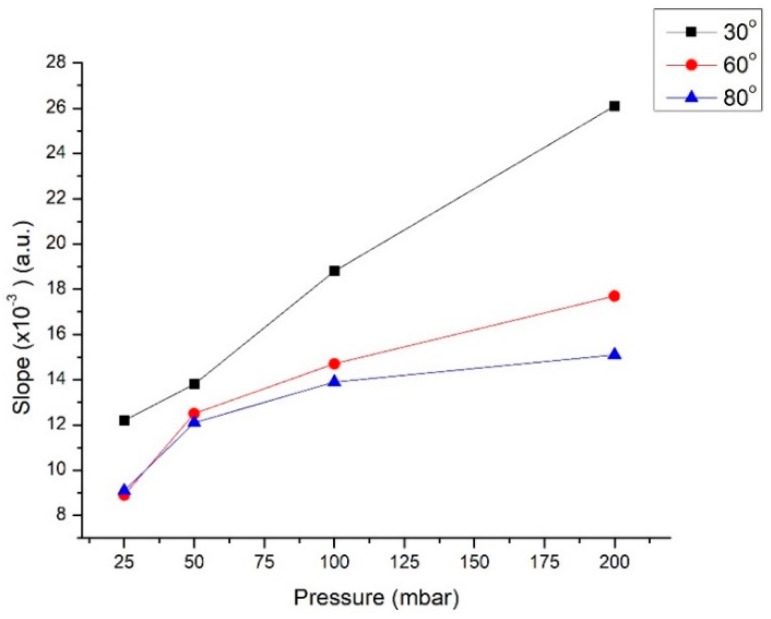
Slope of the linear interpolation of the intensity profile of the visual diffusion analysis (see Figure 3c) in the chip as a function of the pair inlet pressure at the different separation angles *θ* = 30°, 60°, and 80°.

We studied the effect of the angle between the two inlets. All the microfluidic chips were fabricated with a constant chamber height (*h* = 500 μm). Three different angles *θ* = 30°, 60°, and 80° were chosen and the results are reported in Figure 5. Angles greater than 80° exhibited significant diffusion due to the trajectory of the fluids, so we excluded them for the purposes of this work.

For each angle value, the slope of the intensity profile in the diffusion region increases monotonously with the (identical) pumping pressure at the two inlets: the greater the pressure, the greater the velocity of the fluids, leading to an increased value of the Pe coefficient and reduced diffusive mixing. It is clear that for a given pumping pressure, a smaller separation angle results in reduced mixing between the two parallel flowing fluids.

Increasing the separation angle of the inlets, the fluids undergo active mixing in the first part of the chamber due to a greater change in trajectory upon entering the main chamber. In the case of 60° and 80°, a saturation of the slope value is observed. However, in the case of smaller angles, the parallel flows enter into the chamber with minimum turbulence. For this reason, we determined that a separation angle of *θ* = 30° is the most suitable for minimum diffusion flow.

### 3.5. The Effect of Chamber Height

**Figure 6:**
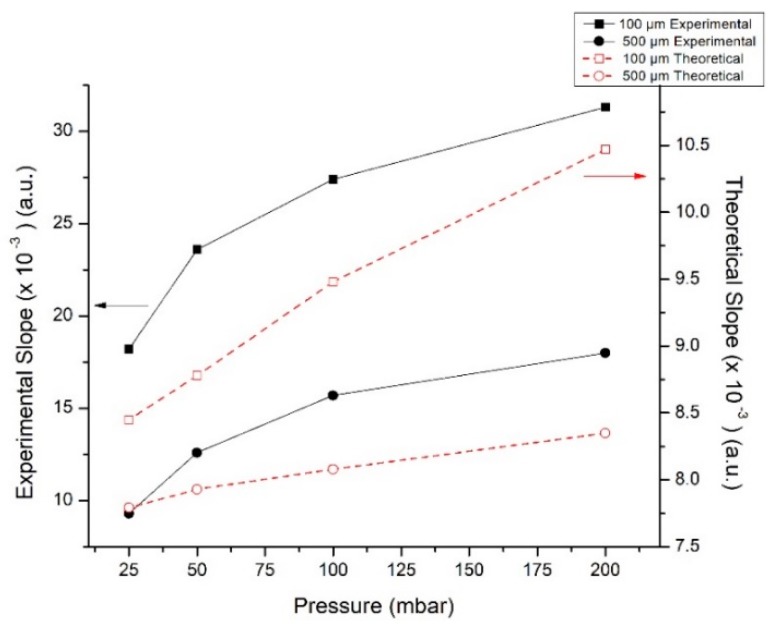
Theoretical and experimental slope of the intensity profile in the diffusion region versus the pumping pressure for two different chamber heights: 100 μm and 500 μm.

In Figure 6, we report the slope of the diffusion zone for channel heights of *h* = 100 μm and 500 μm for chips with a length of 2 mm and an angle of 30° between inlets. It is evident that the channel height has a significant impact on the diffusion process in this microfluidic platform, with the slope in the diffusion region for the 100 μm tall chamber being twice that of the 500 μm tall channel in any pressure measured. In other words, the diffusion is less pronounced in the shorter chamber, irrespective of the pumping pressure. A reasonable explanation for this observation is that by decreasing the height of the chamber, the interaction area of the two fluids decreases as well. Considering the length of the chamber is 2 mm, the interaction area of the fluids is 0.2 mm^2^ for the 100 μm high chamber and 1 mm^2^ for the 500 μm.

Another parameter that justifies this drastic change in the slope is the fluid velocity, which in the case of *h* = 500 μm is much lower than that of *h* = 100 μm (Table 1) at constant pressure. As mentioned before, the diffusion is proportional to the residence time of the interacting fluids in the chamber and consequently inversely proportional to the flow velocity.

The parametric study of the effect of the height the diffusion was simulated by COMSOL, as well as the theoretical data, are also presented in Figure 6. For the purposes of this study, we assumed the same mean velocities as in Table 1. Considering the diffusion coefficient of R6G in ethanol, we were able to extract the slope of the concentration gradient, following the same procedure as for the experimental data.

Although the same general trends are observed in the theoretical simulations, there is a discrepancy in the absolute values of slopes. The resistance of the chip and the roughness of the walls were neglected for the purposes of the theoretical study, which can explain the lower diffusive mixing that is predicted by the simulations compared to the experimental data in Figure 6.

## 4. Conclusions

In this work, we performed a parametric study of the geometry of a double Y-shaped microfluidic chip in order to minimize the diffusive mass transfer between two laminar flows. This first approach on the characterization of such chips examines the behavior of two ethanolic solutions, but the results can be translated in any kind of application of multifunctional complex microfluidic systems with a similar configuration. The optical technique that was developed for the purposes of this study is simple, straightforward, and can be replicated using widely used and easily accessible equipment such as an optical microscope with a CCD camera and image processing software.

The FLICE manufacturing technique has enabled the fabrication of complex 3D geometries of buried microfluidic chips in glass while avoiding the problematic process of sealing two substrates together by welding. Being able to use glass as a substrate for microfluidics removes the limitations that are created by the incompatibility of the majority of elastomers, such as PDMS, and allows for a wider variety of applications in flow chemistry.

For the visualization of the diffusion, a R6G solution and a transparent solution were used, enabling the detection of mixing by a simple optical microscopy image analysis. As a result, we determined that an angle of 30° or lower between the two inlet streams is optimal for non-mixing flow. Also, we found that the height of the interaction chamber has a major impact on the diffusion, with the smaller height of 100 μm being preferable. In future work, we will perform chemical reactions at the interface of the parallel laminar flows in the laser-inscribed buried branching network.

## Figures and Tables

**Table 1:** Calculated flow rates, velocities, Reynolds number (Re), and Peclet number (Pe) for different pumping pressures. ${\bar{\upsilon }}_{1}\;\mathrm{and}\;{\bar{\upsilon }}_{2}\;$ correspond to the flow velocity of glass chips with heights of 100 μm and 500 μm, respectively.

Δ*P* (mbar)	*Q* (μL/min)	${\bar{\upsilon }}_{1}\;(\mathrm{mm}/s)$	${\bar{\upsilon }}_{2}\;(\mathrm{mm}/s)$	Re	Pe
25	13.5	22.5	4.5	2	7507.5
50	27.0	45.0	9.0	3	15,015.0
100	54.1	90.0	18.0	6	30,030.0
200	108.1	180.0	36.0	12	60,060.0
